# Sorafenib maintenance after allogeneic stem cell transplantation in patients with FLT3+ AML receiving midostaurin during induction and consolidation: a retrospective analysis

**DOI:** 10.3389/fonc.2024.1441254

**Published:** 2024-09-19

**Authors:** Giuseppe Sapienza, Marta Castronovo, Stefania Tringali, Roberto Bono, Cristina Rotolo, Antonino Mulè, Valeria Calafiore, Caterina Patti, Cecilia Agueli, Valentina Randazzo, Alessandra Santoro, Domenica Matranga, Luca Castagna

**Affiliations:** ^1^ Bone Marrow Transplantation (BMT) Unit, AOR Villa Sofia-Vincenzo Cervello, Palermo, Italy; ^2^ Onco-Hematology Unit, AOR Villa Sofia-Vincenzo Cervello, Palermo, Italy; ^3^ Onco-Hematology and Cell Manipulation Laboratory Unit, AOR Villa Sofia-Vincenzo Cervello, Palermo, Italy; ^4^ Department of Health Promotion, Mother and Childcare, Internal Medicine and Medical Specialties, University of Palermo, Palermo, Italy

**Keywords:** acute myeloid leukemia (AML), relapse, sorafenib, allogeneic stem cell transplantation (Allo-SCT), maintenance

## Abstract

**Introduction:**

Acute myeloid leukemia (AML) relapse is the main cause of death after allogeneic stem cell transplant (allo-SCT). In AML FLT3+, it was shown that Sorafenib used as maintenance therapy after allo-SCT, significantly reduces the risk of relapse and death.

**Methods:**

We analyzed 29 adult patients with FLT3m AML and underwent allogeneic stem cell transplant from 2019 to 2023. All patients received midostaurin plus conventional CT during induction and consolidation. After transplantation, Sorafenib maintenance was administered in all patients independently from MRD status at transplantation.

**Results:**

Sorafenib maintenance was applied in 18 patients out 29 patients (62%). Median time to start sorafenib was 100 days (range 37-225) and median duration of treatment was 775 days (range 140-1064). For the whole population (n=29), 2-year OS, LFS, and CIR was 76%, 68% and 28%, respectively. The median time to relapse was 137 days (range 49-246). For patients treated with sorafenib (n=18), the 2-year OS, LFS, and CIR were 94%, 84% and 11%, respectively. For the whole population, the 100-day NRM was 0% and 1-year NRM was 3%. Death was caused by transplant-associated thrombotic microangiopathy in 1 patient. For patients who were administered with Sorafenib, the 1-y NRM was 5%. Death was caused by transplant associated transplant-associated thrombotic microangiopathy.

**Discussion:**

This retrospective study suggests that sorafenib maintenance seem to be effective even in patients pre-treated with midostaurin.

## 1 Introduction

Acute myeloid leukemia (AML) is a complex and heterogeneous group of hematologic malignancies characterized by various genetic abnormalities. The FMS-like tyrosine kinase 3 mutation (FLT3m) is recognized to confer a poor prognosis due to a high relapse rate and low survival. FLT3 mutations are present in approximately 30% of newly diagnosed adult AML patients, with internal tandem duplication (ITD) in the juxtamembrane domain being the most common FLT3m. First-line conventional treatment with FLT3 inhibitors (FLT3i), such as midostaurin in association with standard chemotherapy, is considered the gold standard (1). Consolidation with allogeneic stem cell transplantation (allo-SCT) is frequently performed in FLT3m patients to reduce the risk of disease relapse (2). Despite the therapeutic advances with allo-SCT, the risk of disease relapse persists, prompting the exploration of additional treatment strategies. Sorafenib is a first-generation type II FLT3i that has been found to be effective in blocking multiple pathways. It has been shown to be effective in reducing the incidence of relapse after allo-SCT in various retrospective and randomized phase 2 and 3 trials (3–6). In these studies, most of the patients did not receive FLT3i during the induction and consolidation phases.

In our analysis, we have specifically focused on patients who were treated with midostaurin during the conventional treatment phase and subsequently received maintenance therapy with sorafenib following allo-SCT.

## 2 Materials and methods

In this retrospective study, we analyzed adult patients diagnosed with FLT3m AML who underwent allogeneic stem cell transplantation between 2019 and 2023. At diagnosis, Nucleophosmin (NPM1) and FLT3-ITD identification was performed by PCR amplification using gene-specific primers followed by capillary electrophoresis, according to Noguera et al. (7). All patients received midostaurin plus conventional chemotherapy (CT) during induction and consolidation. Pre-transplant conditioning regimens were carefully selected based on the individual needs of patients, ranging from myeloablative to reduced intensity, and in some cases, non-myeloablative. Graft-versus-host disease (GVHD) prophylaxis varied depending on donor type. Patients who received allografts from Human leucocyte antigen (HLA) identical sibling (SIB) or matched unrelated donor (MUD) also received prophylaxis with cyclosporine (CSA), methotrexate (MTX), and anti-thymocyte globulin (ATG), whereas those with haploidentical (HAPLO) and mismatched unrelated donors (mMUD) received a regimen of post-transplantation cyclophosphamide in combination with CSA and mycophenolate mofetil (MMF). Patients who did not receive frontline midostaurin as a part of induction were excluded from the study.

The patients’ minimal residual disease (MRD) status was assessed before transplant through PCR amplification using NPM1 as a molecular marker, and in those without this molecular marker, MRD status was evaluated by multiparametric flow cytometry assay.

In our protocol, sorafenib was administered at a starting dose of 200 mg twice daily according to the SORMAIN trial (4). Sorafenib maintenance was administered in all patients independently from MRD status at the time of transplantation. The time to start sorafenib varied depending on engraftment, presence of acute GVHD, or infectious complications. Sorafenib maintenance was planned for up to 2 years after transplantation.

The primary endpoint was leukemia-free survival (LFS) at 2 years post-transplant. LFS is defined as the time to relapse and/or death. The secondary endpoints were cumulative incidence of relapse (CIR), overall survival (OS), and non-relapse mortality (NRM).

For LFS analysis, the Kaplan–Meier method and log-rank test were used. CIR and NRM were based on cumulative incidence estimates and gray tests. All data were recorded in an Excel data sheet, and analysis was performed using the NCSS 2019 software.

## 3 Results

This study identified 29 patients diagnosed with FLT3m AML who were treated with midostaurin and received allo-SCT. The median age was 55 years (range: 19–73). Patient characteristics are reported in **Table 1**. A total of 24 patients (82%) were in first complete remission (CR1), three patients (10%) were in CR2, and two were not in CR. A total of 22 patients (76%) were MRD-negative for NPM1 or FLT3-ITD. Donor type was SIB for eight patients (28%), MUD/mMUD for 11 (37%), and HAPLO for 10 (34%). The median follow-up was 33 months (range: 5–52).

**Table 1:** Patient, disease, and transplant characteristics.

	Overall (n = 29)	SOR (n = 18)
Age median (range)	54 years (19–73)	55 (31–73)
Mutational status: FLT3-ITD NPM1	29 (100%) 18 (62%)	18 (100%) 9 (56%)
Disease status at ALLO: CR1 CR2 Active	24 (82%) 3 (10%) 2 (10%)	17 (95%) / 1 (5%)
MRD status at ALLO (only CR): Negative (mol) Positive	22 (81%) 5 (18%)	16 (94%) 1 (6%)
Donor: SIB MUD mMUD HAPLO	8 (28%) 10 (34%) 1 (3%) 10 (34%)	4 (22%) 7 (39%) 1 (6%) 6 (34%)
Source: PBSC BM	24 (83%) 5 (17%)	14 (78%) 4 (22%)
Conditioning: MAC RIC	26 (90%) 3 (10%)	17 (95%) 1 (5%)

SOR, sorafenib; MRD, minimal residual disease; CR, complete remission; SIB, sibling; MUD, matched unrelated donor; mMUD, mismatched unrelated donor; HAPLO, haploidentical; PBSC, peripheral blood stem cells; BM, bone marrow; MAC, myeloablative conditioning; and RIC, reduced-intensity conditioning.

The median time to absolute neutrophil count (ANC) of more than 0.5/L and that of platelet count of more than 20/L was 17 days (range: 9–31) and 15 days (range: 9–33), respectively.

In the whole cohort, the cumulative incidence rates of acute GVHD (aGVHD) were 23% and 3% for grades 2–4 and grades 3–4, respectively. The median time to aGVHD was 43 days (range: 25–172). The chronic GVHD (cGVHD) all-grade incidence rate was 15%.

Sorafenib maintenance was administered in 18 out of 29 patients (62%). Reasons for not starting maintenance were as follows: lack of approval by authorities (n = 1, 3%), early relapse (n = 2, 6%), prior tyrosine kinase inhibitor (TKI) resistance (n = 2, 6%), acute GVHD (n = 3, 10%), poor performance status (n = 1, 3%), and other causes (n = 2, 6%). The median time to start sorafenib was 100 days (range: 37–225), and the median duration of treatment was 775 days (range: 140–1,064).

Sorafenib was withdrawn in 12/18 (67%) patients in seven because of the end of treatment, in two because of relapse on therapy, and in three because of complications such as transplant-associated thrombotic microangiopathy (one), idiopathic thrombocytopenic purpura (one), and pericarditis (one).

**Figure 1:**
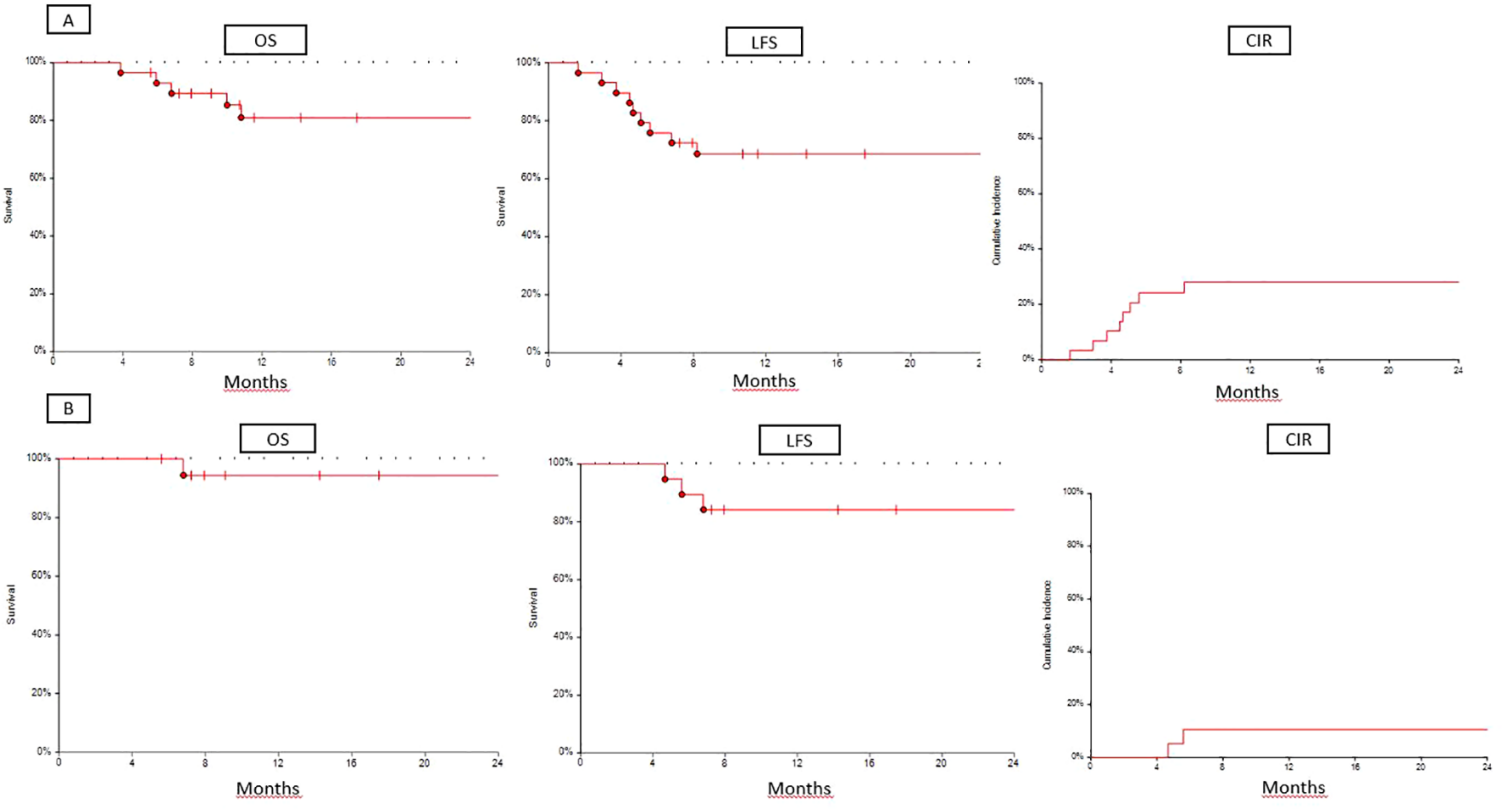
**(A)** OS, LFS, and CIR for whole cohort (n=29). **(B)** OS, LFS, and CIR for sorafenib cohort (n=18).

For the whole population (n = 29), 2-year OS, LFS, and CIR were 76%, 68%, and 28%, respectively. The median time to relapse was 137 days (range: 49–246). For patients treated with sorafenib (n = 18), the 2-year OS, LFS, and CIR were 94%, 84%, and 11%, respectively (**Figure 1**).

For the whole population, the 100-day NRM was 0%, and the 1-year NRM was 3%. Death was caused by transplant-associated thrombotic microangiopathy in one patient. For patients who were administered with sorafenib, the 1-year NRM was 5%. Death was caused by transplant-associated thrombotic microangiopathy.

In the no-sorafenib cohort (n = 10), there were no cases of toxic death; 1-year LFS and OS were 40% and 50%, respectively. Six patients relapsed, and five of them died. Six of ten (60%) patients were CR1/CR2-MRD negative, 3/10 (30%) patients were CR1/CR2-MRD positive, and 1/10 (10%) patients had an active disease. Of the patients who relapsed, three were CR1/CR2-MRD negative, and three were CR1/CR2-MRD positive.

In the sorafenib cohort (n = 19), 16 (84%) patients were CR1-MRD negative, two (11%) patients were CR1-MRD positive, and one (5%) patient had an active disease.

For all patients in CR1 (n = 24), 2-year OS, LFS, CIR, and NRM were 81%, 71%, 25%, and 4%, respectively. The median time to relapse was 140 days (range: 49–204). For patients in CR1 treated with sorafenib (n = 18), the 2-year OS, LFS, CIR, and NRM were 94%, 83%, 11%, and 6%, respectively, and the median time to relapse was 168 (range: 140–204).

## 4 Discussion

Our retrospective analysis suggests that in patients with FLT3-ITD AML receiving midostaurin, sorafenib maintenance after allo-SCT still reduces the incidence of relapse, improving survival. Adding midostaurin to conventional chemotherapy improved the survival of patients with AML with FLT3 mutation (1) and is regularly being used in clinical practice. Not only midostaurin but also quizartinib plus conventional chemotherapy at diagnosis has recently been shown to improve survival in FLT3-ITD-positive patients in a randomized clinical trial (8).

Although allo-SCT is considered mandatory for FLT3-mutated AML in the first complete remission (9), relapse remains a cumbersome point. Several strategies can be used to reduce the risk of relapse, but targeted molecules may be preferred based on a good safety profile. In randomized clinical trials, sorafenib administered as maintenance after allo-SCT significantly improved survival because of the reduction of relapse (4, 5), even when the duration of maintenance was 2 years (5) and 6 months (4). Recently, Xuan et al. updated the results after a median follow-up of 60.4 months, confirming that all major endpoints were improved in the sorafenib arm compared to placebo (10). However, in both of these randomized studies, few patients received midostaurin or any other TKIs during the conventional phase of treatment. In Xuan’s trial, 24% of patients received sorafenib during induction (4), and in the multivariate analysis, this did not impact survival or relapse (9). In the SORMAIN study, only nine patients were pre-treated with midostaurin (5). Based on this, we analyzed patients with FLT3-ITD AML who were treated with midostaurin during induction and consolidation, allo-SCT, and sorafenib as maintenance. Not all eligible patients received sorafenib (60%), primarily due to complications such as acute GVHD (10%). The median time to start maintenance was 100 days, longer than 30 days (median, range: 30–42) in the Chinese trial (4) and between 60 and 100 days in the SORMAIN study (5). Despite this, the results observed in this small cohort (n = 29) of patients were interesting (2-year OS was 75%, 2-year LFS was 70%, and 2-year CIR was 24%) and similar to those reported in randomized studies. Furthermore, considering only patients treated with sorafenib, as expected, OS and LFS were higher than those of the whole cohort (2-year OS and 2-year LFS were 93%, and CIR was 11%). These results strongly suggest that sorafenib is active in patients previously exposed to midostaurin.

Recently, the data from the MORPHO study were published showing that gilteritinib used as maintenance after allo-SCT did not improve relapse-free survival in the whole population but only in those patients who were MRD-positive before allo-SCT. In this trial, approximately 60% of patients received an FLT3 inhibitor during induction/consolidation, and gilteritinib seemed to be more effective in terms of relapse-free survival and OS when FLT3 inhibition was used previously (11).

## 5 Conclusions

This study had several limitations, such as its retrospective nature, small number of patients, time to start sorafenib being longer than that in randomized studies, and patient selection bias. The majority of our patients were in the CR1- and MRD-negative before allo-SCT. Nevertheless, the clinical results seem to be similar to those of other studies (4, 5).

In conclusion, prior exposure to TKIs does not reduce the protective activity of sorafenib administered after allo-SCT in patients with FLT3m AML.

Therefore, our results encourage the potential use of sorafenib as maintenance therapy in the post-transplant setting, offering a promising approach to improve survival and reduce relapse in this patient population.

## Data Availability

The raw data supporting the conclusions of this article will be made available by the authors, without undue reservation.
